# The Preparation of ZnFe_2_O_4_ from Coal Gangue for Use as a Photocatalytic Reagent in the Purification of Dye Wastewater via the PMS Reaction

**DOI:** 10.3390/ma19010169

**Published:** 2026-01-02

**Authors:** Mingxian Zhang, Jinsong Du, Xuemei Zheng, Aiyuan Ma

**Affiliations:** 1 School of Civil Engineering and Urban Planning, Liupanshui Normal University, Liupanshui 553004, China; zhangmingxian@lpssy.edu.cn; 2 School of Chemistry and Materials Engineering, Liupanshui Normal University, Liupanshui 553004, China

**Keywords:** peroxymonosulfate, ZnFe_2_O_4_, simulated sunlight, RhB, coal gangue

## Abstract

The widespread application of Rhodamine B (RhB) poses a serious threat to the aquatic environment. ZnFe_2_O_4_, as a catalyst material, can effectively activate persulfate (PMS) and respond to visible light, thus effectively degrading RhB with the joint assistance of sunlight and PMS. This study recovered Fe_2_O_3_ from high-iron coal gangue through an activating–acid leaching–extracting–back-extracting process and synthesized ZnFe_2_O_4_ catalysts (CG-ZFO) using coal gangue back-extraction liquid as the Fe source by a hydrothermal method and cetyltrimethylammonium bromide (CTAB)-assisted hydrothermal method. The characterization results of X-ray diffraction (XRD), scanning electron microscopy (SEM), and diffuse reflectance spectroscopy (DRS) showed that the CG-ZFO has a pure crystal phase, and the addition of CTAB can effectively improve the photoelectric performance of the catalyst. The synthesized CG-ZFO can produce a significant synergistic effect with simulated sunlight (SS) and PMS, and the constructed SS/CG-ZFO/PMS system had a good degradation effect on RhB. Based on the conclusions of free radical-quenching experiments, electron paramagnetic resonance (EPR) spectroscopy, and X-ray photoelectron spectroscopy (XPS), the main active species in the SS/CG-ZFO/PMS system was identified as _1_O^2^, and the degradation mechanism of RhB was elucidated. CG-ZFO prepared from coal gangue holds promising potential for application in the remediation of organic dye wastewater, and this study also provides a new approach for the resource regeneration of high-iron coal gangue.

## 1. Introduction

Rhodamine B (RhB) is an artificially synthesized organic dye, which is widely used in industries such as textiles, fireworks, and biology [1]. Rhodamine B is a persistent environmental pollutant, with its residues detectable in both surface water and the effluent from sewage treatment plants. The discharge of wastewater into natural drainage channels can cause severe damage to ecosystems, trigger water-related diseases, and even lead to human fatalities. In recent years, considerable attention has been focused on dye pollutants that are difficult to degrade and exert negative environmental impacts [2]. It is therefore crucial to develop effective methods for removing Rhodamine B from wastewater prior to its discharge, in order to mitigate its environmental impacts [3]. Traditional degradation methods for RhB have issues like long time consumption, high cost, and potential for secondary pollution [4]. Advanced Oxidation Processes (AOPs) are a category of chemical treatment techniques that degrade organic pollutants in water through strong oxidative radicals [5]. Persulfate (PMS) is one of the most commonly used oxidants in AOPs. It can be activated to produce strong oxidative radicals like SO_4_˙^−^ and ·OH to degrade various organic pollutants [6]. Light energy, as an environmentally friendly PMS activation technique, has received widespread attention in recent years [7]. However, the most commonly used ultraviolet-light activation technology faces challenges such as high energy consumption, poor penetration, and low utilization rate [8]. Although sunlight has broader applicability and lower cost, its activation efficiency is relatively low. The introduction of a sunlight-responsive photocatalyst within the sunlight/PMS system can significantly amplify the activation efficiency of PMS, produce a large number of strong oxidative radicals in a short time, and achieve efficient, rapid, and thorough degradation of RhB without introducing other pollutants except sulfate ions [9,10,11,12,13].

ZnFe_2_O_4_ is a ferrite material with a spinel structure, excellent electromagnetic properties, good chemical stability, high catalytic activity, and low eddy current loss, which is widely used in water treatment, organic synthesis, battery manufacturing, aerospace, and electronic communication [14,15]. In addition to the above characteristics, ZnFe_2_O_4_, as an n-type semiconductor material responsive to visible light, has attracted significant attention in the field of photocatalysis. It possesses a relatively narrow band gap (1.9~2.1 eV) and can be rapidly excited by UV-Vis light (<700 nm) to generate electrons and holes [16]. The relatively high magnetism and stable catalytic performance further expand the application scope of ZnFe_2_O_4_. Despite the advantages of ZnFe_2_O_4_ as a photocatalyst, such as good chemical stability, a narrow band gap, and high magnetism, the rapid electron-hole recombination rate of ZnFe_2_O_4_ results in a photocatalytic efficiency significantly lower than traditional photocatalysts (ZnO, TiO_2_, etc.) [17]. To improve the photocatalytic efficiency of ZnFe_2_O_4_, surfactants can be added during synthesis to improve the morphological structure and particle size distribution, thereby exposing more photocatalytically active crystal planes [18]. Moreover, studies have found that the presence of oxidants like PMS can capture the photo-generated electrons from ZnFe_2_O_4_, effectively reducing the electron-hole recombination rate of zinc ferrate and enhancing the photocatalytic efficiency of the system [19]. Currently, ZnFe_2_O_4_ is mainly synthesized through sol–gel, solution combustion, and hydrothermal methods using chemicals containing Fe and Zn [20,21,22].

High-iron coal gangue typically refers to coal gangue with an Fe_2_O_3_ content exceeding 8 wt%. The presence of excess Fe_2_O_3_ impurities can greatly affect the resource regeneration of the rich SiO_2_ and Al_2_O_3_ components in the coal gangue [23,24,25]. Acid leaching is commonly employed to pre-treat such coal gangue, achieving the removal and separation of Fe_2_O_3_ and improving the grade [26]. However, adopting this method also generates a large amount of leaching waste solution. Due to the complex metal element composition in the leaching solution, it is difficult to directly use it as a raw material for producing various products. Usually, the pH value is adjusted to precipitate and separate elements such as Fe, Al, and Mg, achieving their separation and recovery [27,28]. This method has drawbacks when dealing with complex element systems like coal gangue leaching solution, including stringent operating conditions, poor selectivity, low separation degree, and impure products.

In this study, high-iron coal gangue was employed as the raw material, and an efficient separation of the Fe_2_O_3_ component was achieved via a sequential process consisting of mechanical ball milling, high-temperature calcination, acid leaching, extraction, and back-extraction. Subsequently, the coal gangue back-extracted solution was combined with an additional Zn source to synthesize a pure-phase ZnFe_2_O_4_ catalyst via the hydrothermal method. The structure and photoelectric properties of the synthesized CG-ZFO were characterized using X-ray diffraction (XRD), scanning electron microscopy (SEM), Fourier transform–infrared spectroscopy (FT-IR), diffuse reflectance spectroscopy (DRS), and i-t transient photocurrent tests. RhB was used as the degradation target, and the SS/CG-ZFO/PMS catalytic system was constructed to perform photocatalytic degradation experiments. The synergistic action between SS, PMS, and CG-ZFO was thoroughly studied, as were the effects of the addition of the surfactant CTAB, PMS dosage, and initial pH value on the degradation efficiency of CG-ZFO. Finally, the degradation mechanism of the SS/CG-ZFO/PMS system was studied through radical-quenching experiments, XPS, and EPR.

## 2. Experimental Section

### 2.1. Experimental Materials

#### 2.1.1. Chemicals

Rhodamine B (C_28_H_31_ClN_2_O_3_, RhB) and methanol (CH_3_OH, MeOH) were purchased from Tianjin Kemiou Chemical Co., Ltd. (CHN) (Tianjin, China). Zinc chloride (ZnCl_2_) was purchased from Meryer (Shanghai) Chemical Technology Co., Ltd. (CHN) (Shanghai, China). Hydrochloric acid (HCl) and sulfuric acid (H_2_SO_4_) were purchased from Chongqing Chuandong Chemical (Group) Co., Ltd. (CHN) (Chongqing, China). Tributyl phosphate (C_12_H_27_O_4_P, TBP) was purchased from Aladdin reagent (Shanghai) Co., Ltd. (CHN) (Shanghai, China). Sulfonated kerosene and peroxymonosulfate (KHSO_4_·0.5KHSO_4_·0.5K_2_SO_4_, PMS) were purchased from Shanghai Yien Chemical Technology Co., Ltd. (CHN) (Shanghai, China). L-histidine (C_6_H_9_N_3_O_2_, L-His) and p-benzoquinone (C_6_H_4_O_2_, BQ) were purchased from Shanghai Macklin Biochemical Co., Ltd. (CHN) (Shanghai, China). Sodium thiosulphate (Na_2_S_2_O_3_) and sodium hydroxide (NaOH) were purchased from Tianjin Zhiyuan Chemical Reagent Co., Ltd. (CHN) (Tianjin, China). Tertiary butyl alcohol (C_4_H_10_O, TBA) was purchased from Tianjin Fuchen Chemical Reagent Co., Ltd. (CHN) (Tianjin, China). All chemicals in this work were analytical reagents (ARs) and used directly without further purification.

#### 2.1.2. Coal Gangue Raw Materials

The coal gangue used in the experiment comes from the Dahebian Coal Mine in Liupanshui, which has the characteristics of a high silicon–aluminum ratio and high iron content. The chemical composition of the coal gangue was shown in Table 1, with an Fe_2_O_3_ content of 20.6% and a certain amount of other impurity components.

### 2.2. Acid Leaching and Extraction of Coal Gangue

The coal gangue was ball milled to pass through a 200-mesh sieve and calcined at 500 °C for 1 h. Afterward, the activated coal gangue was leached under an HCl concentration of 20%, leaching temperature of 90 °C, leaching time of 3 h, and liquid–solid mass ratio of 4: 1 to obtain a coal gangue leaching solution. The leaching solution was collected multiple times and heated to concentrate. The additional HCl was added until the acid concentration of the concentrated solution reached 15%. TBP extractant (with the sulfonated kerosene as the diluent) was used to extract Fe^3+^ in the solution under conditions of 40% TBP concentration, O/A = 2:1 (oil phase/aqueous phase), 20 °C extraction temperature, and 10 min extraction time. Deionized water was used to back-extract Fe^3+^ in the organic phase under conditions of O/A = 1:1, 20 °C extraction temperature, and 10 min extraction time. The above extracting–back-extracting process was repeated twice (during which the TBP extractant could be reused), finally obtaining the coal gangue back-extraction solution.

### 2.3. Preparation of Catalysts

A certain amount of ZnCl_2_ was added to the coal gangue back-extraction solution to make nZn^2+^:nFe^3+^ = 1:2 (molar ratio), and deionized water was added to form a 50 mL homogeneous solution. The solution was heated to 60 °C, and NaOH solution (2 mol·L^−1^) was added dropwise to adjust the pH value to 12 while stirring. The mixed suspension was heated to 80 °C and kept for 1 h, then transferred to a 100 mL Teflon-lined autoclave and kept at 180 °C for 12 h. Finally, the product was filtered and washed with deionized water and ethanol and dried at 80 °C for 12 h to form the CG-ZFO_1_ catalyst. An additional CTAB was added to the homogeneous solution to make nZn^2+^:nFe^3+^:nCTAB = 1:2:4, and the CG-ZFO_2_ catalyst was synthesized using the same steps above.

### 2.4. Experimental Procedures

The 30 mg of CG-ZFO catalyst was added to 100 mL of RhB solution (20 mg·L^−1^) and then placed in a self-built photochemical reaction apparatus for dark adsorption at 20 °C. After the adsorption equilibrium was reached, a certain amount of PMS was added, and the xenon lamp was turned on to initiate the reaction. A 0.1 mol·L^−1^ solution of H_2_SO_4_ and NaOH was used to adjust the initial pH value of the RhB solution. During the reaction process, a 3 mL sample was taken every 20 min. After separating the catalyst using 0.22 μm filter membranes, 3 mL of Na_2_S_2_O_3_ solution was added to terminate the reaction. The concentration of RhB was measured at a wavelength of 554 nm using a UV-Vis spectrophotometer (TU-1901, CHN, Beijing, China). The degradation rate of RhB was calculated by Equation (1):

(1) $$R=\frac{{C-C}_{0}}{C_{0}}\times 100\%$$

where R (%) represents the degradation rate; C_0_ (mg·L^−1^) represents the initial concentration of RhB; and C (mg·L^−1^) represents the concentration of RhB at a specific moment.

### 2.5. Characterization and Analysis Methods

The phase of the catalysts was characterized using a Rigaku Ultima IV X-ray powder diffractometer (XRD, Tokyo, Japan) with Cu Kα radiation (50 kV, 30 mA), and the scanning angle ranged from 10° to 80°. The structure of the catalysts was analyzed using a Fourier transform–infrared spectrometer (FT-IR, Nicolet iS50, Madison, WI, USA). The surface morphology of the catalysts was characterized by a scanning electron microscope (SEM, ZEISS Sigma 300, GER, Oberkochen, Germany) equipped with energy-dispersive X-ray spectroscopy (EDS, Smartedx, GER, Oberkochen, Germany). Using a UV-Vis spectrophotometer equipped with an integrating sphere (UV-Vis DRS, Shimadzu UV-3600, JPN, Kyoto, Japan), the UV–visible diffuse reflectance spectroscopy (DRS) of the catalysts was detected using BaSO_4_ substrate as the background. Na_2_SO_4_ (0.5 mol·L^−1^) was used as the electrolyte solution; the photoelectric properties of the catalysts were measured by a three-electrode cell with a 300 W xenon lamp (with filter-to-filter UV light) as a light source. X-ray photoelectron spectroscopy (XPS, Thermo Scientific K-Alpha, Waltham, MA, USA) was used to analyze the elemental composition and valence state of the catalyst. The elemental composition of coal gangue and coal gangue leaching residue was determined by X-ray Fluorescence (XRF, Rigaku Supermini 200, JPN, Tokyo, Japan). The concentration of ions in the solution was determined by inductively coupled plasma–optical emission spectroscopy (ICP, Agilent 5110, Santa Clara, CA, USA). Electron paramagnetic resonance spectroscopy (EPR, Bruker EMXplus-6/1, GER, Berlin, Germany) was used to detect and confirm active species during the reaction process.

## 3. Results and Discussion

### 3.1. Resource Utilization Process of Coal Gangue

**Figure 1:**
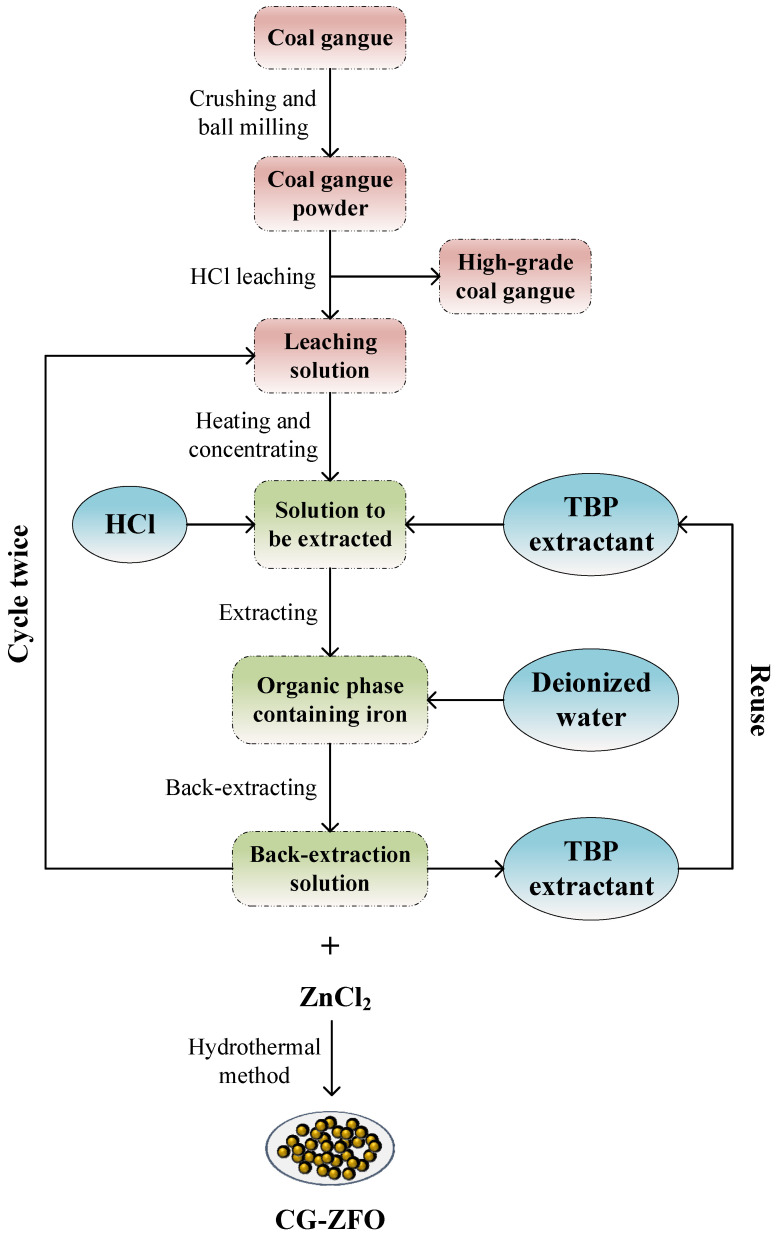
Flowchart of coal gangue resource utilization.

The flowchart for the preparation of CG-ZFO from high-iron coal gangue is shown in Figure 1, with the main steps including mechanical ball milling, high-temperature calcination, HCl leaching, TBP extraction, and deionized water back-extraction.

To remove the Fe_2_O_3_ component from the coal gangue, mechanical ball milling and high-temperature calcination were used for effective activation, followed by leaching under the action of HCl [29]. The chemical composition of the coal gangue leaching residue is shown in Table 2. After acid leaching, most of the impurity components, such as Fe_2_O_3,_ in the coal gangue were removed, thereby facilitating the enrichment and recovery of the SiO_2_ and Al_2_O_3_ components in the coal gangue. Additionally, tributyl phosphate (TBP) was used to extract Fe^3+^ from the coal gangue leaching solution, and deionized water was used for back-extraction, which increased the Fe content in the leaching solution from 46.29% to 99.64%, achieving the separation and purification of Fe elements in the coal gangue (Table 3) [30]. Through the above process, the Fe_2_O_3_ component in the coal gangue was fully recovered and regenerated, and the grade of high-iron coal gangue was simultaneously improved.

### 3.2. Catalyst Characterization

**Figure 2:**
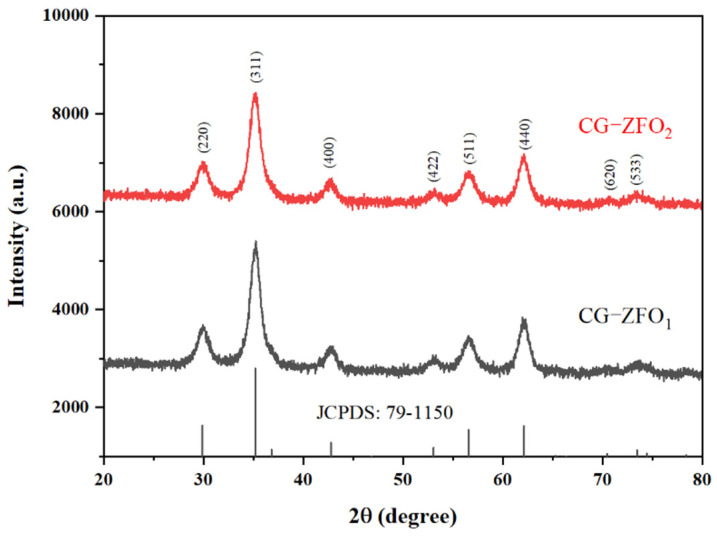
XRD pattern of CG-ZFO catalysts.

Figure 2 shows the XRD pattern of CG-ZFO synthesized using coal gangue back-leaching solution as the Fe source. The complete characteristic peaks corresponding to the (220), (311), (400), (422), (511), (440), and (553) planes (JCPDS: 79-1150) can be observed in both the conventionally synthesized CG-ZFO_1_ and the CTAB-assisted-synthesized CG-ZFO_2_, indicative of the successful synthesis of pure-phase ZnFe_2_O_4_ [31]. Compared with CG-ZFO_2_, the characteristic peaks of CG-ZFO_1_ are sharper, which is due to the absence of surfactant coating on the particle surface to restrict grain growth [32]. According to the Scherrer equation and the main diffraction peak (2θ = 35.211°) value, the average crystallite sizes of CG-ZFO_1_ and CG-ZFO_2_ are calculated to be 8.7 nm and 7.6 nm, respectively. In summary, pure-phase ZnFe_2_O_4_ crystals with spinel structure were successfully synthesized using a coal gangue back-leaching solution, and the addition of CTAB can effectively reduce the crystallite size of the product.

**Figure 3:**
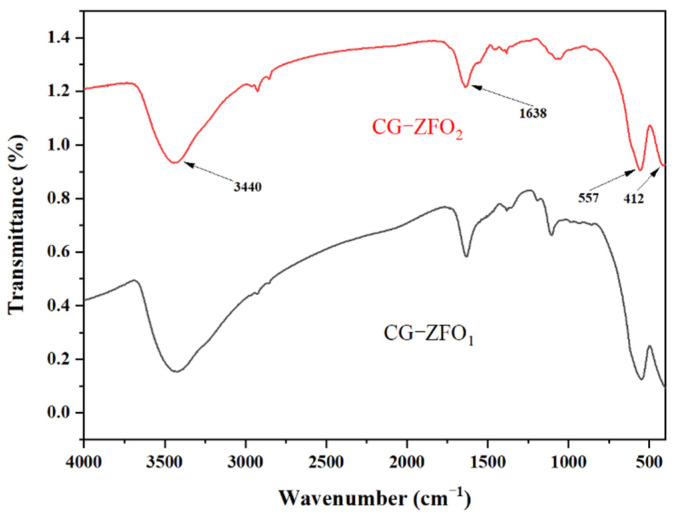
The FT-IR spectra of CG-ZFO catalysts.

Figure 3 shows the FT-IR spectra of CG-ZFO_1_ and CG-ZFO_2_. Through comparison, it was found that the two catalysts have similar peak shapes, representing that their internal structures are basically the same. The broad bands at 3440 cm^−1^ and 1638 cm^−1^ correspond to the stretching and bending vibrations of hydroxyl groups adsorbed on the material surface, which may be due to the hydrothermal reaction. The spectral band appearing at 557 cm^−1^ can be identified as the stretching vibration of the Zn-O bond in the tetrahedral Zn^2+^, and the spectral band appearing at 412 cm^−1^ can be identified as the stretching vibration of the Fe-O bond in the octahedral Fe^3+^ [33]. This result indicates the successful formation of the ZnFe_2_O_4_ crystal structure in the CG-ZFO_1_ and CG-ZFO_2_, which is consistent with the characterization results of XRD.

**Figure 4:**
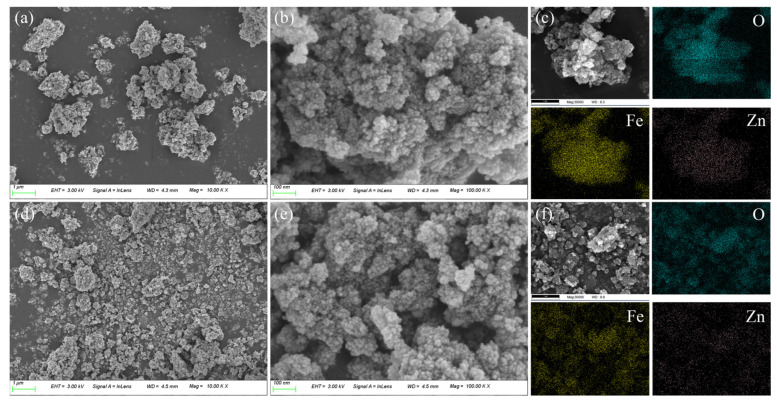
SEM and EDS mapping images of CG-ZFO catalysts, (**a**–**c**) CFO_1_, (**d**–**f**) CFO_2_.

The SEM and EDS mapping images of CG-ZFO are shown in Figure 4, Figure 4a,b show the microscopic surface morphology of CG-ZFO_1_, and Figure 4d,e show that of CG-ZFO_2_. Both catalysts displayed large-particle morphology resulting from the agglomeration of numerous small grains. CG-ZFO_1_ exhibited heterogeneous particle sizes, irregular shapes, and a highly rough surface. In contrast, CG-ZFO_2_ particles were smaller and more uniformly distributed, and exhibited a more regular overall shape. This is because the cationic surfactant CTAB quickly adsorbs on the surface of CG-ZFO_2_ seeds to form an electric double layer at the initial stage of the hydrothermal process, reducing the surface energy of the grains and effectively inhibiting their growth and agglomeration. In addition, the micelles, vesicles, and other aggregates formed by CTAB in the solution also play the role of a soft template during the particle-coating process, controlling the growth and accumulation direction of the grains [34]. Figure 4c,f are the EDS mapping images of CG-ZFO_1_ and CG-ZFO_2_, and the results show that Zn, Fe, and O elements can be evenly distributed in both catalysts.

**Figure 5:**
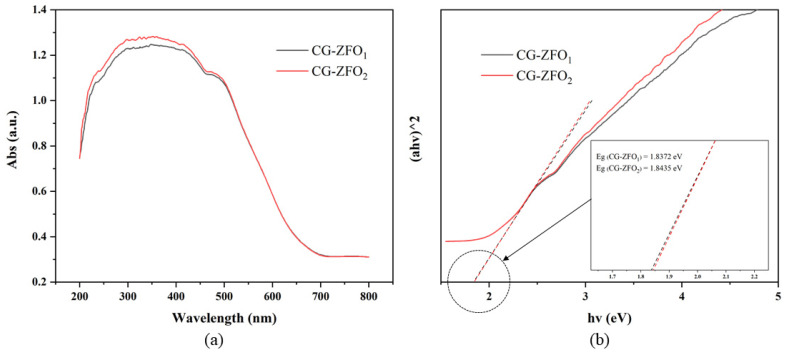
DRS spectra (**a**) and Tauc spectra (**b**) of CG-ZFO catalysts.

Figure 5a presents the UV-Vis diffuse reflectance spectroscopy (DRS) for CG-ZFO_1_ and CG-ZFO_2_. From the DRS, it can be observed that CG-ZFO_2_ exhibited stronger light absorption capabilities within a certain wavelength range. Figure 5b represents the Tauc plot, derived from the Kubelka–Munk transformation of the DRS spectral data [35]. The band gaps of CG-ZFO_1_ and CG-ZFO_2_ are 1.8372 eV and 1.8435 eV, respectively, exhibiting a negligible difference. In summary, the addition of CTAB had a minor effect on the band gap but significantly enhances the light absorption capability of the catalyst.

**Figure 6:**
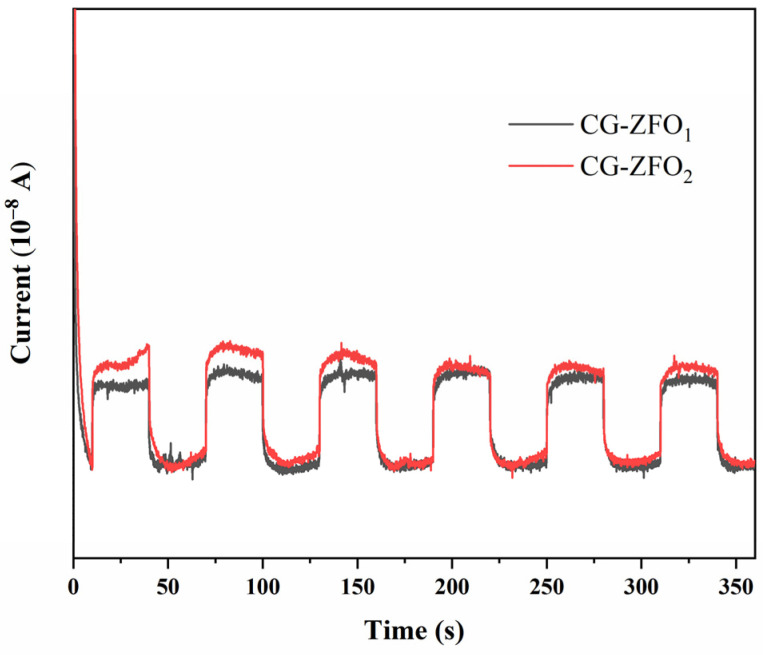
Transient photocurrent response of CG-ZFO catalysts.

Transient photocurrent tests were repeatedly used to analyze the photoelectrochemical dynamic behavior of semiconductor materials. The photoelectric performance of a semiconductor material was evaluated by analyzing the changes in photocurrent generated by the material under the cyclic on–off illumination of the light source [36]. The i-t photocurrent curves for CG-ZFO_1_ and CG-ZFO_2_ are shown in Figure 6. Both CG-ZFO_1_ and CG-ZFO_2_ exhibit significant photocurrent signals instantaneously under visible-light irradiation, and both their photocurrent transient shapes were nearly perfect rectangles. This indicates that the photocurrent signals of the catalysts were very stable, and the separation of photogenerated electrons and holes was good. Notably, CG-ZFO_2_ exhibited a stronger photocurrent signal during the light-on stage, indicating superior visible-light absorption capability and photo-generated charge separation capability. This was attributed to the addition of CTAB, which resulted in smaller and more uniform grain sizes of CG-ZFO and effectively inhibited large-scale agglomeration between particles. This was beneficial for exposing more photocatalytically active crystal planes per unit irradiation area of CG-ZFO, thereby enhancing the photoelectric performance.

### 3.3. Catalytic Performance Study of CG-ZFO Catalysts

#### 3.3.1. Effects of CTAB Addition on RhB Degradation

**Figure 7:**
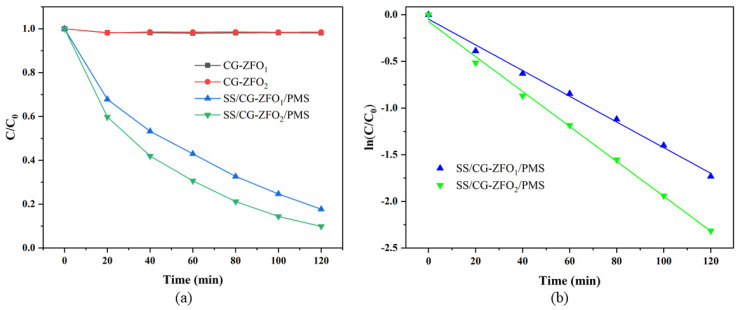
(**a**) Degradation curve and (**b**) degradation kinetics fitting diagram of CG-ZFO_1_ and CG-ZFO_2_. Experimental conditions: (Catalyst, 0.3 g·L^−1^, PMS, 0.5 g·L^−1^, RhB, 20 mg/L, Initial pH = 4.48, Temperature = 20 °C).

Figure 7a shows the difference between CG-ZFO_1_ and CG-ZFO_2_ in terms of adsorption and degradation performance for RhB. It can be seen that the adsorption capabilities of the two catalysts can be basically ignored, and both can reach adsorption equilibrium within 40 min. The SS/CG-ZFO_1_/PMS system can degrade RhB by 82.31% within 2 h, and the SS/CG-ZFO_2_/PMS system can degrade RhB by 90.12% within 2 h. CG-ZFO_2_ exhibited superior catalytic performance compared to CG-ZFO_1_, attributable to its stronger light absorption capability and photo-generated charge separation capability. This result was consistent with the DRS and i-t test results. By fitting the degradation curve, it was found that the degradation process of RhB conformed to the first-order kinetic model (kt = −ln(C/C_0_)). Figure 7b is the degradation kinetics fitting diagram of the SS/CG-ZFO_1_/PMS and SS/CG-ZFO_2_/PMS systems, and the degradation kinetic constants are shown in Table 4. The reaction kinetic constant of the SS/CG-ZFO_1_/PMS system was 0.0138 min^−1^, and the reaction kinetic constant of the SS/CG-ZFO_2_/PMS system was 0.0187 min^−1^. The addition of surfactants increased the degradation rate of the CG-ZFO catalyst by 35.51%. Consequently, all subsequent experiments employed the CG-ZFO_2_ catalyst.

#### 3.3.2. Effects of Catalytic Systems on RhB Degradation

**Figure 8:**
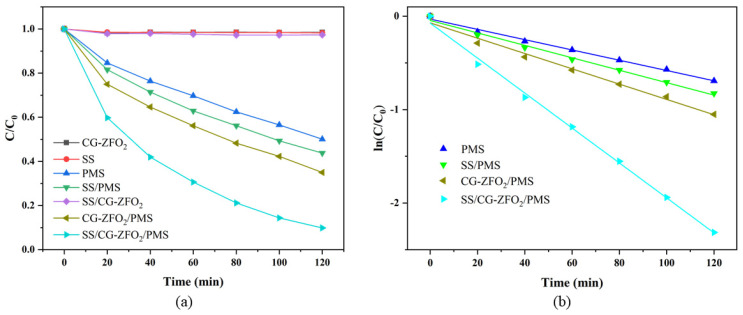
Degradation curve (**a**) and degradation kinetics fitting diagram (**b**) of different catalytic systems. Experimental conditions: (Catalyst, 0.3 g·L^−1^, PMS, 0.5 g·L^−1^, RhB, 20 mg/L, Initial pH = 4.48, Temperature = 20 °C).

In order to study the roles of the CG-ZFO_2_, SS, and PMS in the degradation process of RhB, seven catalytic systems, including CG-ZFO_2_, SS, PMS, SS/PMS, SS/CG-ZFO_2_, CG-ZFO_2_/PMS, and SS/CG-ZFO_2_/PMS, were constructed for catalytic degradation experiments. As shown in Figure 8a, the concentration of RhB solution under SS irradiation only had a slight change (about 2%), indicating that RhB exhibited good chemical stability under light conditions. Similarly, RhB was also essentially undegraded in the SS/CG-ZFO_2_ system, primarily due to the rapid electron-hole recombination rate of ZnFe_2_O_4_. PMS can self-activate in water to produce the strong oxidizing radical SO_4_˙^−^; hence, the PMS system exhibited a considerable degradation effect, achieving a 50% degradation of RhB within 2 h. The introduction of SS and CG-ZFO_2_ into the PMS system can significantly accelerate the degradation rate of RhB. The SS/PMS catalytic system can degrade RhB by 56.23% within 2 h, and the CG-ZFO_2_/PMS catalytic system can degrade RhB by 65% within 2 h. Among all catalytic systems, the SS/CG-ZFO_2_/PMS system had the best degradation effect, and this system can degrade RhB by 90.12% within 2 h. The degradation efficiency of the SS+PMS+CG-ZFO_2_ catalytic system for RhB degradation is also compared with other compounds reported in the literature, listed in Table 5.

Figure 8b is the degradation kinetics fitting diagram of the PMS, SS/PMS, CG-ZFO_2_/PMS, and SS/CG-ZFO_2_/PMS catalytic systems, with the reaction kinetic constants shown in Table 6. The kinetic constant of the SS/CG-ZFO_2_/PMS system surpasses the sum of the kinetic constants of the SS/PMS system and the CG-ZFO_2_/PMS system, indicating that the high degradation efficiency of the SS/CG-ZFO_2_/PMS system was not merely a simple addition of SS, CG-ZFO_2_, and PMS, but rather, they exhibit significant synergistic effects.

#### 3.3.3. Effects of Initial pH on RhB Degradation

**Figure 9:**
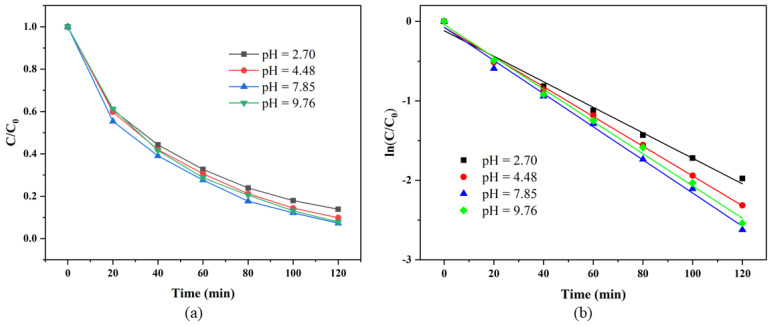
Degradation curve (**a**) and degradation kinetics fitting diagram (**b**) at different initial pH values. Experimental conditions: (Catalyst, 0.3 g·L^−1^, PMS, 0.5 g·L^−1^, RhB, 20 mg/L, Temperature = 20 °C).

Figure 9a shows the effect of the initial pH on the degradation efficiency of the SS/CG-ZFO_2_/PMS system. The SS/CG-ZFO_2_/PMS system exhibited good degradation performance within a wide pH range of 2.7 to 9.76. Specifically, within the pH range of 2.7 to 7.85, the degradation rate of RhB escalated with increases in pH. However, when the pH value exceeded 9.76, the degradation rate of RhB began to decline. The kinetics fitting diagram of the degradation curves under different initial pH values is shown in Figure 9b, with the corresponding reaction kinetic constants enumerated in Table 7. From a kinetics perspective, within the pH range of 2.7 to 7.85, the reaction kinetic constant increased from 0.0161 min^−1^ to 0.0209 min^−1^. Further increasing the pH value caused the reaction kinetic constant to decrease to 0.0203 min^−1^. The main reason for the lower degradation rate of RhB in an acidic environment was the high concentration of H^+^ in the solution, which quenched the free radicals (mainly SO_4_˙^−^ and ·OH) in the solution (Equations (2) and (3)) [42].

·OH + H^+^ + e^−^ → H_2_O (2)

SO_4_˙^−^ + H^+^ + e^−^ → HSO_4_˙^−^ (3)

Therefore, as the concentration of H^+^ in the solution significantly decreased, the quenching probability of free radicals in the solution greatly reduced, and the degradation efficiency of RhB significantly improved. When the pH value reached 9.76, the degradation rate of RhB began to decrease. This was because when the pH value reached above 8, the SO_4_˙^−^ in the solution began to react with H_2_O or OH^−^ to produce ·OH with a lower redox potential (Equations (4) and (5)) [43].

SO_4_˙^−^ + H_2_O → SO^2−^ + ·OH + H^+^ (4)

SO_4_˙^−^ + OH^−^ → SO^2−^ + ·OH (5)

In addition, when the pH value reached above 9.4, the form of PMS in the solution changed from HSO_5_^−^ to SO_5_^2−^, which also affected the generation of active species and thus reduced the degradation efficiency of RhB [44].

#### 3.3.4. Effects of PMS Dosage on RhB Degradation

**Figure 10:**
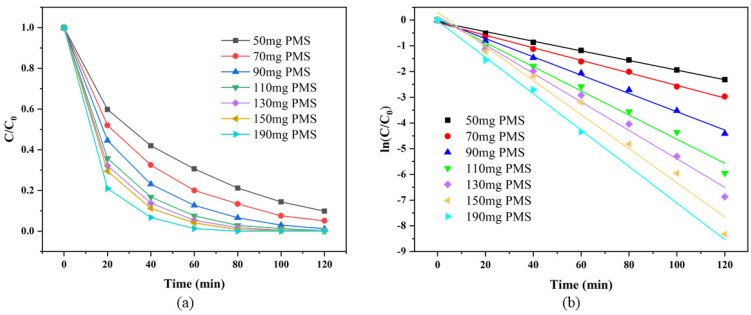
Degradation curve (**a**) and degradation kinetics fitting diagram (**b**) under different PMS dosages. Experimental conditions: (Catalyst, 0.3 g·L^−1^, RhB, 20 mg/L, Initial pH = 4.48, Temperature = 20 °C).

The effect of PMS dosage on the degradation efficiency in the SS/CG-ZFO_2_/PMS system is shown in Figure 10a. As the PMS dosage increased, the degradation rate of RhB obviously rose. This was because the more PMS was added, the higher the concentration of free radicals produced by activation. Overall, within the range of 50 to 190 mg, the addition of PMS always played a positive role in the degradation of RhB in the SS/CG-ZFO_2_/PMS system. However, as the PMS dosage gradually increased, the effect on enhancing the degradation rate of RhB exhibited a gradually weakening trend. Figure 10b is the kinetic fitting diagram of the degradation curves under different initial pH values, and the specific reaction kinetic constants are shown in Table 8. It can be found that within the range of 50 to 110 mg PMS dosage, each increase of 20 mg PMS dosage gradually increased the reaction kinetic constant of the system by 30.36%, 45.76%, and 32.04%, while within the range of 110 to 150 mg PMS dosage, each increase of 20 mg PMS dosage only gradually increased the reaction kinetic constant of the system by 17.98% and 19.57%. When the PMS dosage in the system reached 150 mg, an increase of 40 mg PMS dosage only increased the reaction kinetic constant of the system by 6.97%. This suggests that in the SS/CG-ZFO_2_/PMS system, when the PMS dosage reached a certain amount, the catalytic efficiency was significantly inhibited. This was mainly because the excessive HSO_5_^−^, SO_4_˙^−^, ·OH in the solution produced self-quenching reactions, producing SO_4_^2−^, HSO_4_^−^ with no degradation ability or SO_5_˙^−^ with weak degradation ability (Equations (6)–(8)) [45].

SO_4_˙^−^ + SO_4_˙^−^ → 2SO_4_^2−^ (6)

·OH + HSO_5_^−^ → SO_4_˙^−^ + H_2_O (7)

SO_4_˙^−^ + HSO_5_^−^ → SO_5_˙^−^ + HSO_4_^−^ (8)

### 3.4. Degradation Mechanism Study

#### 3.4.1. Identification of Active Species

**Figure 11:**
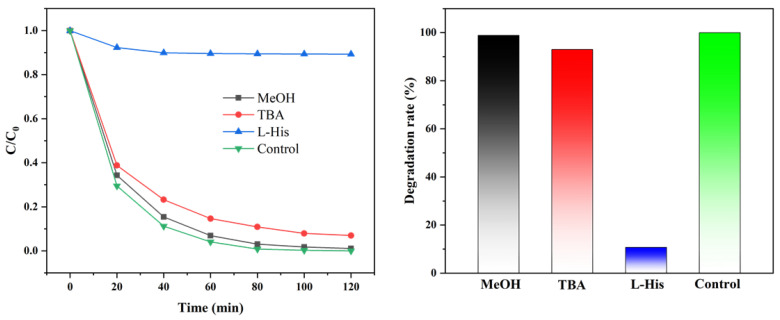
Free radical-quenching experiment in SS/CG-ZFO_2_/PMS system. Control conditions: (Catalyst, 0.3 g·L^−1^, PMS, 1.5 g·L^−1^, RhB, 20 mg/L, Initial pH = 4.48, Temperature = 20 °C).

A radical-quenching experiment was used to explore the main active species in the SS/CG-ZFO_2_/PMS catalytic system. The study showed that in the SS/CG-ZFO_2_/PMS catalytic system, SO_4_˙^−^, ·OH, and ^1^O_2_ are important active species in the reaction [46,47]. The reaction rate of methanol (MeOH) with SO_4_˙^−^ is 1.1 × 10^7^ M^−1^S^−1^, and the reaction rate with ·OH is 2.5 × 10^8^ M^−1^S^−1^, while the reaction rate of tert-butanol (TBA) with SO_4_˙^−^ is 4.0∼9.1 × 10^5^ M^−1^S^−1^, and the reaction rate with ·OH is 3.8∼7.6 × 10^8^ M^−1^S^−1^ [48]. Therefore, in this experiment, MeOH (500 mM) was used as a quencher for both SO_4_˙^−^ and ·OH, and TBA (500 mM) was used as a quencher for ·OH. The respective contributions of SO_4_˙^−^ and ·OH to the reaction system were determined by comparing the degradation rate changes between two sets of experiments. The reaction rate between L-histidine (L-His) and ^1^O_2_ is 1.2 × 10^8^ M^−1^S^−1^; hence, L-His (10 mM) was used as a quencher for ^1^O_2_ [49]. The final experimental results are shown in Figure 11. L-His had the most significant inhibitory effect on the degradation in the SS/CG-ZFO_2_/PMS catalytic system. At 100 min of reaction time, the presence of L-His reduced the degradation rate of RhB from 100% to 10.63%, indicating that ^1^O_2_ was the main active species in the SS/CG-ZFO_2_/PMS catalytic system. Additionally, at 100 min of reaction time, TBA reduced the degradation rate of RhB from 100% to 92.08%, and MeOH reduced it to 98.21%, indicating that SO_4_˙^−^ and ·OH also played a certain role in the reaction system. It is worth noting that the inhibitory effect of MeOH on the SS/CG-ZFO_2_/PMS catalytic system was significantly lower than that of TBA, which may be due to the fact that most of the active radicals in the SS/CG-ZFO_2_/PMS catalytic system were generated on the surface of the CG-ZFO_2_ catalyst, and MeOH, with strong hydrophilicity, had difficulty fully contacting and reacting with the free radicals on the catalyst surface [48].

**Figure 12:**
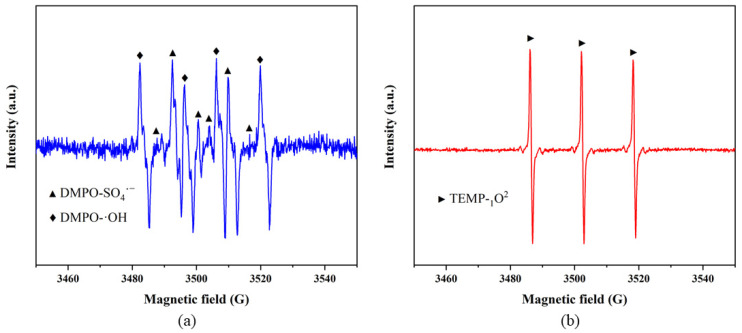
EPR spectra of the SS/CG-ZFO_2_/PMS system with trapping agent (**a**) DMPO, (**b**) TEMP. Experimental conditions: (Catalyst, 0.3 g·L^−1^; PMS, 1.5 g·L^−1^; reaction time, 15 min; DMPO/TEMP, 100 mM; reaction temperature, ambient temperature).

EPR was used to further confirm the active species within the SS/CG-ZFO_2_/PMS system. During the testing process, DMPO (5,5-dimethyl-1-pyrroline N-oxide) was used to capture SO_4_˙^−^ and ·OH, while TEMP (2,2,6,6-tetramethylpiperidine-1-oxyl) was used to capture ^1^O_2_. After adding PMS to the solution containing the catalyst and turning on the xenon lamp for 15 min, significant radical signal peaks can be observed in the EPR spectra. The peaks in Figure 12a belonged to DMPO-SO_4_˙^−^ and DMPO-OH, and the peaks in Figure 12b belonged to TEMP-^1^O_2_ [50]. This conclusion fully confirms the generation of sulfate radicals (SO_4_˙^−^), hydroxyl radicals (·OH), and singlet oxygen (^1^O_2_) during the catalytic process, and this finding is consistent with the results obtained from the radical-quenching experiments.

#### 3.4.2. XPS Analysis

**Figure 13:**
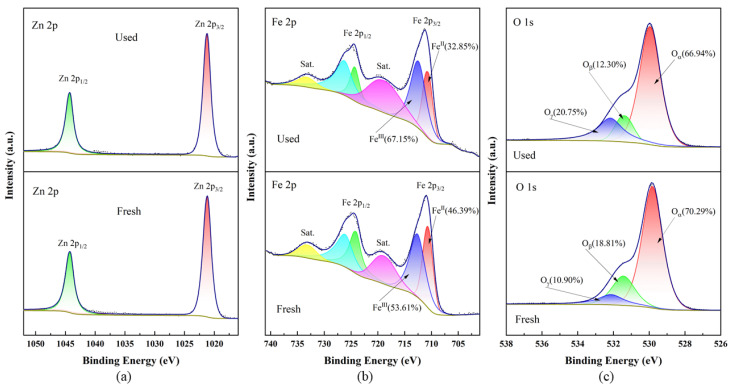
XPS spectra of fresh and used CG-ZFO_2_ catalysts, (**a**) Zn 2p, (**b**) Fe 2p, (**c**) O 1s.

The XPS spectra of the CG-ZFO_2_ before and after the degradation reaction in the SS/CG-ZFO_2_/PMS catalytic system are shown in Figure 13. The binding energies of all elements in the spectra were calibrated using C 1s (284.8 eV) as the reference peak. In the Zn 2p XPS spectra of the fresh CG-ZFO_2_ shown in Figure 13a, two characteristic peaks can be observed at binding energies of 1021.17 eV and 1044.21 eV, which are attributed to the Zn 2p_3/2_ peak and 2p_1/2_ peak in the tetrahedral sites of Zn^2+^ in CG-ZFO_2_ [51]. In the Fe 2p XPS spectra of the fresh CG-ZFO_2_ shown in Figure 13b, four characteristic peaks can be observed at binding energies of 710.98 eV, 718.84 eV, 724.86 eV, and 733.15 eV. The peaks at 710.98 eV and 724.86 eV are attributed to Fe 2p_3/2_ and Fe 2p_1/2_, while the peaks at 718.84 eV and 733.15 eV are attributed to two distinct satellite peaks of Fe 2p. The two peaks at 710.67 eV and 712.63 eV, divided from the Fe 2p_3/2_ characteristic peak, were fitting peaks attributed to Fe^II^ and Fe^III^ [52]. The O 1s spectra of the fresh CG-ZFO_2_ surface are shown in Figure 13c, where the O 1s peak can be divided into three distinct fitting peaks at binding energies of 529.82 eV, 531.44 eV, and 532.14 eV, which were attributed to lattice oxygen (O_α_), surface oxygen (O_β_) (brought by surface oxygen-containing groups), and adsorbed oxygen (O_γ_) (brought by surface-adsorbed H_2_O) [53]. After the degradation reaction, the elemental chemical state of the CG-ZFO_2_ surface underwent significant changes. As can be seen from Figure 13a, the peak shape and position of the Zn 2p_3/2_ and 2p_1/2_ peaks did not basically change in the used CG-ZFO_2_, indicating that Zn did not participate in reactions affecting the valence state of the used CG-ZFO_2_. The relative content of Fe^II^ in the fresh CG-ZFO_2_ catalyst was 46.39%, and the relative content of Fe^III^ was 53.61%. After the degradation reaction occurred, the relative content of Fe^II^ decreased to 32.85%, and the relative content of Fe^III^ increased to 67.15%. This indicates that Fe plays an important role in the degradation reaction of the SS/CG-ZFO_2_/PMS system. In the O 1s fitting peaks of fresh CG-ZFO_2_, the relative content of O_α_, O_β_, and O_γ_ was 70.29%, 18.81%, and 10.9%, respectively. After the degradation reaction occurred, the relative content of O_α_ on the used CG-ZFO_2_ surface decreased to 66.94%, the relative content of O_β_ decreased to 12.3%, and the relative content of O_γ_ increased to 20.75%. This indicates that O_α_ and O_β_ participated in the reaction. The significant increase in O_γ_ is caused by the surface-adsorbed H_2_O formed by the degradation of RhB.

#### 3.4.3. Degradation Mechanism

**Figure 14:**
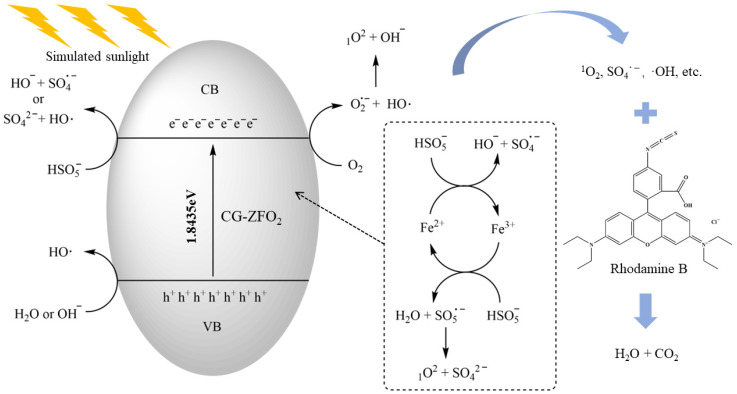
Degradation mechanism of the SS/CG-ZFO_2_/PMS system.

Based on the conclusions of the quenching experiments, EPR, and XPS, a possible degradation mechanism of the SS/CG-ZFO_2_/PMS catalytic system is proposed, as shown in Figure 14. The electron transfer promoted by the redox cycle of Fe^II^ and Fe^III^ on the surface of CG-ZFO_2_ facilitated the activation of PMS in water, resulting in the generation of SO_4_˙^−^ and SO_5_˙^−^ (Equations (9) and (10)) [54]. The SO_5_˙^−^ generated by the above reaction self-quenches to produce ^1^O_2_ (Equation (11)) [55]. In addition, the photo-generated electrons and holes produced by the excitation of CG-ZFO_2_ by simulated sunlight can also participate in the reactions within the system to promote the activation of PMS. The PMS in the solution can be activated by photo-generated electrons to generate SO_4_˙^−^ and ·OH (Equations (13) and (14)) [46]. Moreover, O_2_ adsorbed on the surface of CG-ZFO_2_ can react with photo-generated electrons to generate O_2_˙^−^ (Equation 15), which then reacts with ·OH or H_2_O to produce ^1^O_2_ (Equations (16) and (17)) [56]. OH^−^ and H_2_O can be activated by photo-generated holes to generate ·OH (Equations (18) and (19)) [47]. In summary, the redox cycle of Fe^II^/Fe^III^ and the generation of photo-generated electrons and holes can effectively promote the activation of PMS in the catalytic system, forming a variety of active free radicals with ^1^O_2_ as the main component to achieve the degradation of RhB.

Fe^II^ + HSO_5_^−^ → Fe^III^ + SO_4_˙^−^ + OH^−^ (9)

Fe^III^ + HSO_5_^−^ + H^+^ → Fe^II^ + SO_5_˙^−^ + H_2_O (10)

SO_5_˙^−^ + SO_5_˙^−^ → ^1^O_2_ + 2SO_4_^2−^ (11)

ZnFe_2_O_4_ + hν → e^−^ + h^+^ (12)

e^−^ + HSO_5_^−^ → SO_4_^2−^ + ·OH (13)

e^−^ + HSO_5_^−^ → SO_4_˙^−^ + OH^−^ (14)

O_2_ + e^−^ → O_2_˙^−^ (15)

O_2_˙^−^+ ·OH → ^1^O_2_ + OH^−^ (16)

2O_2_˙^−^ + 2H_2_O → ^1^O_2_ + H_2_O_2_ + 2OH^−^ (17)

h^+^ + OH^−^ → ·OH (18)

h^+^ + H_2_O → ·OH + H^+^ (19)

SO_4_˙^−^/OH/^1^O_2_ + RhB → intermediates → CO_2_ + H_2_O (20)

## 4. Conclusions

This paper used the continuous process of activating–acid leaching–extracting–back-extracting to recover Fe_2_O_3_ from high-iron coal gangue as an Fe source, and synthesized the CG-ZFO catalyst through the hydrothermal method, subsequent to the addition of a Zn source. The CG-ZFO was used for the activation of PMS by the synergistic action of SS to degrade RhB in water. A series of degradation experiments based on CG-ZFO were constructed to clarify the synergistic action between SS, PMS, and CG-ZFO. Furthermore, the influence of CTAB addition, initial pH, and PMS dosage on the degradation efficiency was studied. Finally, based on radical-quenching experiment, EPR, and XPS conclusions, the degradation mechanism of the SS/CG-ZFO/PMS catalytic system is proposed.

1. Through activation and acid leaching processes, Fe_2_O_3_ in the coal gangue was effectively removed. Subsequently, the leaching solution was subjected to extraction using tributyl phosphate (TBP) followed by back-extraction with deionized water. This series of operations increased the iron (Fe) concentration in the solution from 46.29% to 99.64%, making it a feasible iron source for the synthesis of the ZnFe_2_O_4_ catalyst.
2. CG-ZFO was characterized by XRD, SEM, FT-IR, etc., showing that the catalysts have a pure ZnFe_2_O_4_ crystal structure. The addition of CTAB resulted in a smaller crystal grain size, higher dispersion, and better light absorption capability and photo-generated charge separation capability of the catalyst.
3. A series of degradation experiments based on CG-ZFO further confirmed the enhancement of the catalyst’s photoelectric performance by CTAB addition and the synergistic action between SS, PMS, and CG-ZFO. It was found that when the initial pH was 7.85 and the PMS dosage was 150 mg, the degradation performance of the catalytic system was maximized.
4. Combining the conclusions of radical-quenching experiments, EPR, and XPS, it was determined that in the SS/CG-ZFO_2_/PMS catalytic system, ^1^O_2_ was the main active species. The CG-ZFO_2_ catalyst mainly relies on the Fe^II^/Fe^III^ redox cycle and the generation of photo-generated electron-holes to promote the activation of PMS for the degradation of RhB.

In summary, our future research will focus on optimizing the recovery process, enhancing catalyst performance, deepening mechanistic understanding, expanding application scope, and ensuring sustainability. By pursuing these directions, we aim to contribute significantly to the field of environmental catalysis and promote the development of sustainable wastewater treatment technologies.

## Figures and Tables

**Table 1:** Chemical composition of coal gangue. Unit: %.

Composition	SiO_2_	Al_2_O_3_	Fe_2_O_3_	TiO_2_	CaO	K_2_O	MgO	P_2_O_5_	ZrO_2_	Cr_2_O_3_
Content	44.80	17.40	20.60	6.64	3.54	3.11	1.14	0.27	0.22	0.11

**Table 2:** Chemical composition of leaching residue. Unit: %.

Composition	SiO_2_	Al_2_O_3_	Fe_2_O_3_	TiO_2_	CaO	K_2_O	MgO	P_2_O_5_	ZrO_2_
Content	70.90	12.00	2.95	9.82	0.14	2.97	0.42	0.091	0.23

**Table 3:** Element content of leaching solution and back-extraction solution. Unit: %.

Element	Fe	Al	Ca	Mg	K	Na	P	Zn
Leaching solution	46.29	32.16	9.90	6.75	2.84	0.92	0.42	0.10
Back-extraction solution	99.640	0.009	0.0329	0.003	0.085	0.027	0.084	0.120

**Table 4:** Degradation kinetic constants of CG-ZFO_1_ and CG-ZFO_2_.

Catalytic System	k_obs_ (min^−1^)	R^2^
SS/CG-ZFO_1_/PMS	0.0138	0.9939
SS/CG-ZFO_2_/PMS	0.0187	0.9962

**Table 5:** The comparative degradation efficiency of similar catalysts under the different experimental conditions.

No.	Catalyst System	Dye	Irradiation/Time (min)	Degradtion	Ref.
1	SS+PMS+CG-ZFO_2_	Rhodamine B	Xe-lamp 120 min	90.12%	Present work
2	SnO_2_	Rhodamine B	Xe-lamp 120 min	54.7%	[37]
3	ZnFe_2_O_4_	Rhodamine B	Xe-lamp 120 min	32.5%	[37]
4	SnO_2_/ZnFe_2_O_4_	Rhodamine B	Xe-lamp 120 min	72.6%	[37]
5	Co_3_O_4_	Rhodamine B	UV light 240min	61%	[38]
6	ZnFe_2_O_4_	Rhodamine B	UV light 240 min	43%	[38]
7	0.8Co_3_O_4_/0.2ZnFe_2_O_4_	Rhodamine B	UV light 240 min	92%	[38]
8	ZnFe_2_O_4_ nanoparticles	Rhodamine B	UV light 300 min	97%	[39]
9	ZnFe@CuS 25%	Rhodamine B	visible light 150 min	93%	[22]
10	ZnFe_2_O_4_/Bi_2_WO_6_	Rhodamine B	visible light 180 min	93%	[40]
11	ZnFe_2_O_4_-0%@ZnO	Rhodamine B	visible light 240 min	34.62%	[41]
12	ZnFe_2_O_4_-50%@ZnO	Rhodamine B	visible light 240 min	91.87%	[41]

**Table 6:** Degradation kinetic constants of different catalytic systems.

Catalytic System	k_obs_ (min^−1^)	R^2^
PMS	0.0055	0.9927
SS/PMS	0.0067	0.9904
CG-ZFO_2_/PMS	0.0082	0.9864
SS/CG-ZFO_2_/PMS	0.0187	0.9962

**Table 7:** Degradation kinetic constants at different initial pH values.

Initial pH	k_obs_ (min^−1^)	R^2^
2.70	0.0161	0.9888
4.48	0.0187	0.9962
7.85	0.0209	0.9943
9.76	0.0203	0.9950

**Table 8:** Degradation kinetic constants under different PMS dosages.

PMS Dosage (mg)	k_obs_ (min^−1^)	R^2^
50	0.0187	0.9962
70	0.0244	0.9954
90	0.0356	0.9954
110	0.0469	0.9857
130	0.0554	0.9885
150	0.0662	0.9800
190	0.0708	0.9934

## Data Availability

The original contributions presented in this study are included in the article. Further inquiries can be directed to the corresponding authors.
